# Role of tertiary lymphoid structures and B cells in clinical immunotherapy of gastric cancer

**DOI:** 10.3389/fimmu.2024.1519034

**Published:** 2025-01-07

**Authors:** Weiyi Chen, Lingli Zhang, Man Gao, Ning Zhang, Rumeng Wang, Yang Liu, Yan Niu, Lizhou Jia

**Affiliations:** ^1^ Basic Medical Sciences, Inner Mongolia Medical University, Hohhot, Inner Mongolia, China; ^2^ College of Veterinary Medicine, Inner Mongolia Agricultural University, Hohhot, Inner Mongolia, China; ^3^ Bayannur Clinical Medical College, Inner Mongolia Medical University, Hohhot, Inner Mongolia, China; ^4^ Central Laboratory, Bayannur Hospital, Bayannur, Inner Mongolia, China; ^5^ Medical Experiment Center, Inner Mongolia Medical University, Hohhot, Inner Mongolia, China

**Keywords:** gastric cancer, tertiary lymphoid structure, B cells, immunotherapy, clinical application

## Abstract

Gastric cancer is a common malignant tumor of the digestive tract, and its treatment remains a significant challenge. In recent years, the role of various immune cells in the tumor microenvironment in cancer progression and treatment has gained increasing attention. Immunotherapy, primarily based on immune checkpoint inhibitors, has notably improved the prognosis of patients with gastric cancer; however, challenges regarding therapeutic efficacy persist. Histological features within the tumor microenvironment, such as tertiary lymphoid structures (TLSs), tumor-infiltrating lymphocytes, and the proportion of intratumoral stroma, are emerging as potentially effective prognostic factors. In gastric cancer, TLSs may serve as local immune hubs, enhancing the ability of immune cells to interact with and recognize tumor antigens, which is closely linked to the effectiveness of immunotherapy and improved survival rates in patients. However, the specific cell type driving TLS formation in tumors has not yet been elucidated. Mature TLSs are B-cell regions containing germinal centers. During germinal center formation, B cells undergo transformations to become mature cells with immune function, exerting anti-tumor effects. Therefore, targeting B cells within TLSs could provide new avenues for gastric cancer immunotherapy. This review, combined with current research on TLSs and B cells in gastric cancer, elaborates on the relationship between TLSs and B cells in the prognosis and immunotherapy of patients with gastric cancer, aiming to provide effective guidance for precise immunotherapy.

## 1 Introduction

Gastric cancer (GC) is the fifth most common malignant tumor worldwide and the fifth leading cause of cancer-related death, with its incidence and mortality rates surpassing those of other malignant tumors (1). According to Global Cancer Statistics 2022, there were 968,000 new cases of gastric cancer, accounting for 4.9% of all malignant tumors. In the same year, 689,000 deaths were attributed to gastric cancer, constituting 76.8% of cancer-related deaths (1). The popularization of early screening, diagnosis, and treatment has led to a decrease in the mortality rate of patients with gastric cancer (2). However, the early symptoms of gastric cancer are often subtle, and many cases are already at an advanced stage when diagnosed. In recent years, advances in medical research and technology have led to the emergence of new treatment approaches, such as immunotherapy. Immunotherapy works by activating or enhancing the patient’s own immune system to target cancer cells, offering unique therapeutic advantages and potential. Currently, immunotherapy combined with chemotherapy is considered an effective treatment for advanced gastric cancer, with immune checkpoint inhibitor (ICI)-based immunotherapy showing significant effectiveness in reactivating anti-tumor immune responses (3, 4). Nonetheless, there are still patients who exhibit low response rates to immunotherapy (5, 6). Therefore, accurately identifying the beneficiaries of immunotherapy and enhancing its efficacy remains a major challenge.

The tumor microenvironment (TME) is a key factor influencing the efficacy of immunotherapy (7). In addition to tumor cells, it includes immune cells, fibroblasts, and the tumor vascular system, with tertiary lymphoid structures (TLSs) serving as important markers within the TME (8). These structures promote the infiltration of immune cells into solid tumors. TLSs are ectopic aggregations of immune cells that resemble secondary lymphoid organs (SLOs) and are commonly observed in conditions such as chronic inflammation, infectious diseases, autoimmune diseases, transplanted organs, and tumors (9–11). Some studies have shown that the presence of B cells in tumors is a strong prognostic marker, potentially surpassing T cells in prognostic value. Mature TLSs (mTLSs) are structured with the T-cell zone surrounding the B-cell zone, which contains the germinal center, indicating a high concentration of B cells in TLSs. During germinal center formation, B cells undergo affinity maturation, class switching, and somatic hypermutation, developing into B cells with mature immune functions (12, 13).

TLSs have become a research focus in recent years and have been shown to have potential as predictors of immunotherapy response, as well as independent prognostic markers (14). The association between TLSs and tumor immunotherapy efficacy has been observed across various solid tumors, with tumors containing TLSs demonstrating greater sensitivity to immunotherapy (15, 16). In gastric cancer, immune cell clusters with TLSs are present, and studies have shown that a high density of TLSs is associated with improved survival rates (17, 18). However, in the current research on TLSs and B cells in gastric cancer, there are still numerous knowledge gaps.

On the one hand, TLSs and B cells in gastric cancer potentially possess dual roles. They may exhibit both anti-tumor and pro-tumor effects under specific circumstances. Nevertheless, it remains unclear how the balance and switch between promoting anti-tumor and pro-tumor effects occur, as well as the specific mechanisms underlying their influence. Although it has been discovered that B cells are crucial for the antitumor effects of TLSs and are associated with a better prognosis in cancer patients, key questions such as the specific signaling pathways, intercellular interactions, and molecular mechanisms through which B cells play this central role, as well as their manifestations in different gastric cancer subtypes and at various disease stages, remain unanswered. This restricts the effective implementation of a B-cell-centered approach to inducing the formation of TLSs and realizing their antitumor effects in clinical practice. On the other hand, despite studies having identified some prognostic and predictive value in TLSs-related indicators, translating these findings into clinical practice is fraught with difficulties. For instance, the density and maturity of TLSs vary considerably among patients, making it challenging to accurately and conveniently evaluate the TLSs status in each patient using the available assays. Consequently, it is not possible to reliably screen the population that could benefit from immunotherapy based on TLSs-related indicators.

In this review, we will explore the prognostic value and immunotherapy relationship of TLSs in gastric cancer by analyzing the anti-tumor immune response of TLSs and the status and capabilities of B cells, with the intention of uncovering the deficiencies in current studies and furnishing a more all-encompassing and precise research foundation for the immunotherapy of gastric cancer. This would assist in bridging the relevant knowledge gaps and facilitating the translation of research findings into clinical practice.

## 2 Overview of TLSs

### 2.1 Structure and formation of TLSs

SLOs are key components of the immune system, including lymph nodes, tonsils, Peyer’s patches, spleen, and mucosa-associated lymphoid tissue (MALT) (19). SLOs present foreign antigens through antigen-presenting cells (APCs), promoting the activation of B and T cells (20). TLSs are ectopic lymphoid structures formed by lymphocytes in non-lymphoid tissues under pathophysiological conditions (21). They primarily consist of a B-cell zone, a T-cell zone, dendritic cells (DCs), follicular DCs (FDCs), high endothelial venules (HEVs), and germinal centers (22). TLSs share similar structural and functional characteristics with SLOs, and the stromal cells within them function like follicular reticular cells in SLOs, localizing them at sites of chronic inflammation (23). However, unlike SLOs, most TLSs lack a fibrous capsule. The immune cells in TLSs are directly exposed to the surrounding tissues, enabling them to mount a more efficient immune response. This characteristic makes the functions of TLSs more complex and diverse (11, 12) (**Table 1**).

**Table 1:** Comparison of SLOs and TLSs.

	SLOs	TLSs	Refer
Structural composition	Scattered B cell zone and T cell zone, few PCs, macrophages, dendritic cells and endothelial cells.	Change from scattered B cell and T cell zone to aggregated B cell and T cell zone, follicular dendritic cells, macrophages and HEVs.	(198)
Envelope	Enveloped to separate SLOs from its surroundings.	No envelope, TLSs are directly exposed to various factors in the microenvironment.	(199)
Development and formation mechanisms	Formed during embryonic development according to a specific gene regulatory program.	Induced formation in an acquired chronic inflammatory or disease state.	(200)
Location and local microenvironment	Relatively fixed lymphoid organs in the body, such as lymph nodes, spleen, etc.	Temporary lymphoid structures located in non-lymphoid tissues, such as tumor tissues, chronic inflammatory tissues, etc.	(201)
Scope and pertinence	Play the role of immune surveillance and immune defense in the systemic immune system.	Focus on local tissue immunomodulation.	(202)
Functional characteristics	The main sites for the maturation and differentiation of immune cells and the initiation of immune response.	Initiation of a local immune response.	(202)

SLOs, secondary lymphoid organs; TLSs, tertiary lymphoid structures; PCs, plasma cells; HEVs, high endothelial venules.

TLSs are divided into immature TLSs (iTLSs) and mTLSs (24). iTLSs exhibit a disordered aggregation of T cells and B cells, with their interactions promoting both lymphocyte activation and TLS maturation. mTLSs predominantly form structures resembling lymphoid follicles in SLOs that contain germinal centers. These structures are surrounded by a type of HEVs that facilitate the rapid entry of immune cells, such as T and B cells, into TLSs, playing a crucial role in regulating immune responses and enhancing immune surveillance (25, 26).

**Figure 1:**
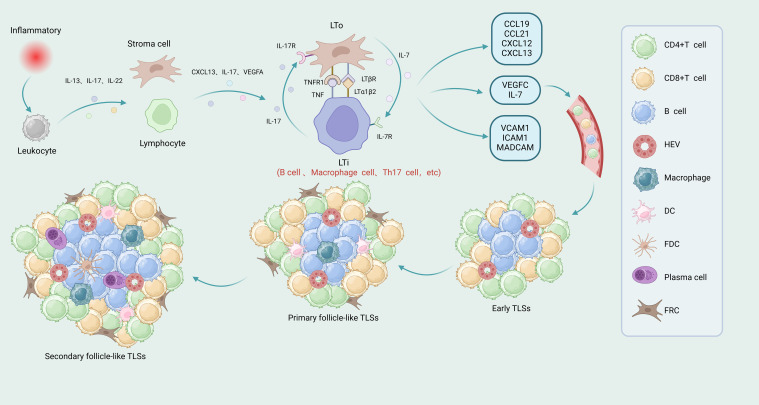
Formation process and maturity stages of TLSs. Under the stimulation of chronic inflammation, leukocytes release IL-13, IL-17 and IL-22, induce the activation of lymphocytes and LTo in tumor tissues, secrete CXCL13, IL-7 and VEGFA, and then induce the recruitment of LTi cells. LTo and LTi may promote the development of TLSs through the interaction of TNF and its receptors TNFR1, LTα1β2 and its receptors LTβR, IL-7 and its receptors IL-7R, IL-17 and its receptors IL-17R. During the interaction process, it will release chemokines CCL19, CCL21, CCL12 and CXCL13 that promote the partition of T cells and B cells, VEGFC and IL-7 that promote the formation of HEVs, and vascular adhesion molecule 1 (VCAM1), intercellular adhesion molecule 1 (ICAM1), and mucosal vascular addressin cell adhesion molecule 1 (MADCAM1) that promote the recruitment of immune cells. The maturation of TLSs can be divided into three stages: early TLSs, primary follicle-like TLSs and secondary follicle-like TLSs. Early TLSs contained T cells, B cells and HEVs. Primary follicle-like TLSs increased DCs, macrophages and FRCs on the basis of early TLSs. Secondary follicle-like TLSs were more mature and had more PCs and FDCs than primary follicle-like TLSs. LTo, lymphoid tissue organizer; LTi, lymphoid tissue inducer; HEVs, high endothelial venules; TLSs, tertiary lymphoid structures; PCs, plasma cells; DCs, dendritic cells; FDCs, follicular dendritic cells; FRCs, fibroblastic reticular cells.

The germinal center, a specialized microstructural region within mTLSs, serves as a critical site for B-cell proliferation, somatic hypermutation, and antibody affinity maturation (27, 28). The formation of TLSs primarily involves three key factors: the induction of lymphotoxin production, the secretion of lymphocyte chemokines, and the generation of HEVs (29) (**Figure 1**). During TLS formation, lymphoid tissue inducer (LTi) cells bind to LTβR on lymphoid tissue organizer (LTo) cells by expressing the lymphotoxin α1β2 ligand (LTα1β2), which promotes the secretion of vascular endothelial growth factor C by LTo cells, mediating the development of HEVs and the secretion of adhesion molecules (8). LTi cells can also bind to receptors on LTo cells by secreting IL-17, which encourages the secretion of chemokines such as CXCL12, CXCL13, CCL19, and CCL21 by LTo cells. These chemokines promote the expression of LTα1β2, subsequently recruiting T and B cells into their respective zones via HEVs (30). CCL21 and CXCL12 are involved in lymphocyte recruitment, whereas CXCL13 and CCL19 work with adhesion molecules to form the structural organization of TLSs (31). When LTi is absent, immune cells such as macrophages, B-lymphocytes, and Th17 cells can replace LTi and interact with stromal cells to induce the formation of TLSs in tumor tissues or at sites of inflammation, and the resulting TLSs exhibit all of the characteristic structures associated with the generation of an adaptive immune response (32).

There are three stages in the maturation of TLSs within the TME: early TLSs, primary follicle-like TLSs, and secondary follicle-like TLSs. Early TLSs contain HEVs but do not contain FDCs or germinal centers. Primary follicle-like TLSs contain both HEVs and FDCs but lack germinal centers. Secondary follicle-like TLSs are more mature, containing HEVs, FDCs, and germinal centers (33) (**Figure 1**).

### 2.2 The differences of TLSs in different gastric cancer subtypes

The different classifications of gastric cancer can reflect the disparities in histology, molecular characteristics, and clinical responses of tumors. Recently, studies have indicated that these differences might be closely related to the properties of TLSs. Intestinal type gastric cancer and diffuse type gastric cancer are the two primary histological types of gastric cancer. Kemi et al. carried out a retrospective cohort study to assess the differences in TLSs among different histological types of gastric cancer (17). High-density TLSs were not associated with the improvement of survival rates in either diffuse or intestinal type gastric cancers. The maximal diameter of TLSs was positively correlated with the survival rate of diffuse type gastric cancer, but had no correlation with that of intestinal type gastric cancer (17).

In accordance with the site of tumor occurrence and the presence of metastasis, gastric cancer can be divided into primary tumors (PTs) and peritoneal metastases (PMs). Recently, in an effort to evaluate the effect of the location and immune context of gastric cancer on TLSs, a research team carried out an analysis of PTs and PMs by means of immunohistochemistry (IHC). Interestingly, they identified that there was no significant difference in the count of TLSs between the two entities (34). The TLSs within PTs displayed a characteristic organizational pattern, with CD20 positioned internally and CD3 externally. In contrast, the TLSs in PMs were chiefly encompassed by cancer cells, stromal cells, and peritoneal adipose tissue (34). By employing spatial transcriptomics to dissect tumor cells and immune cells in PTs and PMs, it was established that in comparison to PTs, the expression of macrophages and regulatory T cells (Tregs) was more pronounced in PMs (34). Additionally, it was noted that the maturity of TLSs was site-specific. Specifically, 70% of the TLSs in PTs were in a mature state, and these mTLSs may potentially have a more substantial role in the anti-tumor immune response (34). Meanwhile, 75% of PMs harbored immature TLSs. It is postulated that this could potentially be a consequence of the modification of the microenvironment in the metastatic loci, leading to a greater prevalence of immunosuppressive cells in the local area. The factors secreted by these cells might impede the maturation of TLSs (34). Alternatively, the gene expression of cancer cells might undergo alterations during the metastatic cascade, thereby inducing changes in the interaction with immune cells and consequently influencing the maturation and function of TLSs. However, the essential etiological factor underlying this difference has yet to be elucidated.

All in all, the differences in the impact of different subtypes of gastric cancer on TLSs are significant. Future research could focus on the interaction mechanisms between the molecular characteristics of different subtypes and TLSs, explore potential intervention targets and treatment approaches, and improve the precision diagnosis and treatment level of gastric cancer.

### 2.3 Evaluation of TLS maturity

Because of the varying definitions and descriptions of TLS maturity markers, TLS density, and germinal centers in tumors, comparing their prognostic value and heterogeneity is challenging. Therefore, it is crucial to establish a unified and standardized approach for determining the role of TLSs in tumors. mTLSs are characterized as network structures containing HEVs, FDCs, and germinal centers (33). The presence of the germinal center serves as a key criterion for TLS maturity, and selecting specific biomarkers is essential for studying TLSs (35).

Traditional histological methods are common means for evaluating the maturity of TLSs, such as hematoxylin-eosin (HE) staining. Through the staining intensity in the cell nucleus, cytoplasm and tissue structure, the morphology and general structure of TLSs can be initially observed. In HE-stained sections, TLSs appear as areas of lymphocyte aggregation with distinct boundaries from surrounding tissues (36). However, HE staining can only predict TLS maturity when visible germinal centers are present, which poses certain limitations (37).

IHC utilizes the principle of specific binding between antigens and antibodies to detect the expression of specific antigens in tissue sections through labeled antibodies. IHC can be used to study the changes in the cellular composition of TLSs under different disease conditions, and it is able to identify the distribution and density of the types of immune cells within TLSs more accurately (38) (**Table 2**). Studies have shown that the expression of CD21 is lower in mTLSs, while the expression of CD23 is higher. When detecting TLSs, labeling CD23 alone can be more sensitive than using both CD20 and CD23 (12, 37). Nevertheless, the precision of the outcomes hinges upon the caliber and specificity of the antibodies. Given that diverse laboratories might employ antibodies with discrepant sources, concentrations, and incubation parameters, such variances could potentially precipitate divergent results. Additionally, IHC is restricted to the detection of pre-known antigens, thereby rendering it incapable of identifying newly unearthed cell markers or antigens associated with uncharted cell functions.

**Table 2:** Relevant biomarkers to assess maturity of TLSs.

	Biomarkers	Functions	Refer
B cell related	CD20	In mTLSs, CD20+ B cells usually form distinct follicular structures surrounded by T cells.	(203)
	PAX5	It can be used to confirm the existence and maturity of B cells, and judge the maturity of TLSs according to the change of PAX5 expression intensity and distribution.	(204)
T cell related	CD3	The distribution and number of CD3+ T cells in TLSs can reflect the involvement of T cells. As the maturity of TLSs increased, the number of CD3+T cells increased and orderly distributed.	(205)
	CD4, CD8	In mTLSs, they coordinate and participate in the immune response together. CD4+/CD8+ ratio reflects the maturity of TLSs.	(206)
Markers of cytokines and chemokines	CXCL13	It is chemotactic for B cells and plays a key role in the formation and maturation of TLSs. High levels of CXCL13 are usually associated with mTLSs.	(67)
	CCL21	It has a chemotactic effect on T cells and DCs, and its expression level correlates with the maturity of TLSs. In mTLSs, CCL21 expression may be higher, helping to attract immune cells into the structure.	(141)
Follicular dendritic cells (FDCs) markers	CD21, CD23, CD35	In mTLSs, FDCs formed a more complete network structure. The functional status of FDCs and the maturity of TLSs can be assessed by detecting the expression of CD21 and CD23.	(203)
HEVs markers	PNAd, MECA79, MAdCAM	It is the specific marker of HEVs and can also be used as the core marker of TLSs.	(207)
Other markers	Ki-67	By detecting the expression of Ki-67, the proliferation status of cells in TLSs can be understood, which indirectly reflects the maturity of TLSs.	(208)
	DC-Lamp	Evaluate the maturity of TLSs by detecting the density of DC lamp.	(209)
	PD-1 and PD-L1	In mTLSs, the interaction between immune cells may lead to changes in the expression of PD-1 and PD-L1. By detecting their expression, the maturity of TLSs can be assessed.	(160)

mTLSs, mature TLSs; DCs, dendritic cells; FDCs, follicular dendritic cells; HEVs, high endothelial venules.

Multiplex immunofluorescence (mIF) is similar to IHC, but it uses fluorescently labeled antibodies. From the perspective of spatial genomics, mIF can detect TLSs at the molecular level, not only displaying the composition of immune cells within TLSs but also accurately identifying different subtypes of immune cells in tumor tissues (39, 40). mIF can be utilized to study complex cell-cell interaction processes such as the formation of immune synapses in TLSs, which has certain advantages. However, fluorescent signals are prone to quenching, and overlapping and interference may also occur, thus affecting the observation results.

Assessment of TLS maturity using IHC and mIF has revealed inconsistencies between the two methods, which may be related to the location of the FDC marker CD23. If CD23 appears outside TLSs but closely adjacent to them, determining TLS maturity becomes challenging (37). At present, there is an IHC assay that uses single staining with CD20 and CD23 simultaneously to detect TLS maturity, ensuring co-localization of CD20+ B cells and CD23+ FDCs cells. This facilitates easy access to samples and offers higher sensitivity compared with staining alone (37). The selection of biomarkers can also lead to disparities between them. To begin with, TLSs encompass a variety of cell types, and each cell might possess multiple potential biomarkers. Currently, there is no standardized biomarker criterion for assessing the maturity of TLSs. For instance, when it comes to the evaluation of B cells, markers like CD20 and CD79a could be chosen. However, the expressions of these markers are likely to vary in diverse diseases and at different maturity levels of TLSs, and they can merely partially mirror the functions and conditions of B cells within TLSs (41). Secondly, the expressions of biomarkers undergo dynamic alterations during tumor progression and the maturation process of TLSs. The levels of TLS-related biomarkers may differ significantly at distinct phases of tumor occurrence. Nevertheless, currently, there is a dearth of efficient methods to monitor such dynamic fluctuations, thereby rendering it arduous to precisely gauge the maturity of TLSs.

Apart from the aforementioned conventional histological assessment techniques, several emerging technologies founded on gene expression or proteomics have progressively emerged and gained prominence. Nevertheless, these technologies have not achieved extensive dissemination as of yet. Moreover, they encounter obstacles in the realms of data interpretation and standardization. It is anticipated that in the days to come, a greater number of novel technologies will assist us in attaining a more profound comprehension of the functions fulfilled by TLSs within tumors.

## 3 Mechanisms of effects of TLSs

### 3.1 Immune regulation

TLSs indeed play a complex dual role in the tumor microenvironment. They may not only promote anti-tumor immune responses but also induce immune tolerance or drive tumor progression under certain circumstances.

Cellular immunity is mainly executed by T cells, with cytotoxic T lymphocytes (CTLs) serving as the central force of this response (42). Although B cells play a relatively minor role in cellular immunity, they can indirectly assist in certain cases. Within TLSs, the presence and activity of Tregs exert a profound influence on the immune equilibrium. Tregs are capable of expressing inhibitory molecules, including CTLA-4 and PD-1, as documented in reference (43). Under specific tumor conditions, TLSs can furnish a conducive milieu for the recruitment and activation of Tregs. Chemokines such as CCL22, which are secreted by stromal cells or other immune cells within TLSs, have the capacity to attract Tregs, as indicated in reference (44). Once Tregs become enriched within TLSs, they can engage in interactions with effector T cells and antigen-presenting cells. Tregs can impede the activation and proliferation of CTLs and helper T cells through both cell-cell contact and the secretion of immunosuppressive cytokines like IL-10 and transforming growth factor-β (TGF-β), as reported in reference (45). This consequently leads to the attenuation of the anti-tumor immune response, the promotion of immune tolerance, and ultimately, the facilitation of tumor progression.

Macrophages within TLSs can clear pathogens or tumor cell debris through phagocytosis and present antigens to T cells, thereby participating in immune activation (46). Additionally, cytokines such as interferon-γ (IFN-γ) secreted by T cells can activate macrophages, transforming them into more active M1 macrophages. This transformation enhances their phagocytic and cytotoxic abilities, contributing to immune defense (47). Nevertheless, within the TME, it is possible for tumor cells to trigger the polarization of macrophages into the M2 phenotype. In the context of TLSs, M2 macrophages are capable of secreting cytokines like IL-10 and TGF-β. These secreted factors not only possess the ability to suppress the activation and functional activities of T cells but also have an impact on the functions of other immune cells residing within TLSs. As a result, the overall immune response is skewed towards immune tolerance (46). Moreover, the M2 phenotype can also promote angiogenesis within TLSs by secreting VEGF, thus providing a favorable environment for tumor growth and metastasis.

B cells are the primary executors of humoral immunity, while helper T cells play a crucial auxiliary role (48). In TLSs, B cells can be activated through direct interaction with T cells and recognition of antigens (49). Upon recognizing specific antigens via B-cell receptors on their surface, B cells internalize, process, and present the antigen to T cells (50). Under the influence of helper T cells, activated B cells further proliferate and differentiate into plasma cells (PCs), which synthesize and secrete large quantities of specific antibodies that exert immune effects in various ways (51). For instance, antibodies can bind directly to antigens on the surface of gastric cancer cells, labeling them for recognition and attack by immune cells, and inducing cell death through complement-dependent cytotoxicity or antibody-dependent cell-mediated cytotoxicity (52).

Moreover, antibodies produced by B cells can regulate the TME by altering the extracellular matrix components surrounding tumor cells, thereby affecting their growth and migratory capabilities (53). Within TLSs, B cells are also capable of facilitating immune tolerance. Regulatory B cells (Bregs) are likely to exist within TLSs. These Bregs have the capacity to generate immunosuppressive cytokines and engage in interactions with T cells (54). They are capable of suppressing the activation and proliferation of T cells, an effect analogous to that of Tregs. Bregs can also exert an impact on the functions of dendritic cells and macrophages, consequently modifying the overall immune response within TLSs. In addition, B cells can secrete cytokines and chemokines, such as IL-21 and CXCL13, which are involved in regulating the recruitment and activation of immune cells, further enhancing the immune response (55, 56).

### 3.2 Regulation of cytokines and chemokines

Cytokines in the TME promote the formation of TLSs. For example, increased concentrations of cytokines such as tumor necrosis factor-α (TNF-α) and IL-1β during inflammation or within the TME can induce the expression of adhesion molecules in vascular endothelial cells, facilitating the recruitment of lymphocytes to local tissues, thereby laying the groundwork for TLS formation (57). Cytokines such as IL-7 stimulate the proliferation and survival of lymphocytes, contributing to their accumulation and maintenance within TLSs (58).

Furthermore, cytokines can regulate the functions of immune cells. For example, IL-2 promotes the proliferation and activation of T cells, enhancing their cytotoxic functions and playing a key role in regulating T-cell immune responses in TLSs (59). Cytokines such as IL-4 and IL-10 are involved in regulating the activation, differentiation, and antibody class switching of B cells, impacting their functionality in TLSs and the types of antibodies produced (60).

Cytokines also influence the maturity of TLSs. For example, IFN-γ promotes the maturation and functional enhancement of FDCs in TLSs, enabling them to present antigens more effectively to B cells, which in turn fosters the formation of germinal centers and the maturation of TLSs (61). Conversely, some cytokines with immunosuppressive effects, such as transforming growth factor-β (TGF-β), may inhibit TLS maturation and the activity of immune cells within the TME (62). TGF-β disrupts the normal activation, differentiation, and antibody production processes of B cells within TLSs. Consequently, B cells are unable to effectively participate in the anti-tumor immune response. Moreover, TGF-β also hinders the maturation and functional optimization of TLSs, thereby weakening the immune surveillance and cytotoxic capabilities of TLSs against tumor cells (63). Furthermore, TGF-β can also promote the epithelial-mesenchymal transition (EMT) of tumor cells. This endows tumor cells with enhanced migratory and invasive capabilities, enabling them to more easily evade the attacks of local immune cells. It thereby accelerates tumor metastasis and dissemination and further facilitates immune evasion (64).

The chemokines CXCL13, CCL19, and CCL21 are highly expressed at TLS formation sites and play a crucial role in promoting immune cell recruitment (31). For example, CXCL13 attracts B cells to migrate toward TLSs by binding to the CXCR5 receptor on their surface, while CCL19 and CCL21 bind to corresponding receptors on T cells, guiding them into TLSs and facilitating the specific recruitment and localization of immune cells (65, 66). Additionally, concentration gradients of different chemokines contribute to the establishment of specific cellular distribution patterns within TLSs. B cells aggregate in areas with high concentrations of CXCL13 to form B-cell follicles, while T cells are distributed in corresponding areas under the influence of CCL19 and CCL21. This orderly cellular distribution is essential for immune cell interactions and the effective initiation of immune responses within TLSs (67–69).

Although cytokines and chemokines are of vital significance in TLSs and the tumor immunity process, there exist a multitude of potential challenges when they are regarded as therapeutic targets. Cytokines and chemokines possess pleiotropic properties, signifying that a single cytokine or chemokine is capable of acting upon diverse cell types and mediating a wide range of biological functions (70). When specific cytokines or chemokines are subjected to targeted interventions, the drugs might interact with unforeseen targets, thereby instigating off-target effects. These off-target effects will not merely undermine the treatment’s efficacy but also have the potential to give rise to a series of adverse side effects, heightening the health risks for patients. Owing to the extreme complexity and sensitivity of the immune system, during the execution of therapeutic interventions on cytokines or chemokines, it is prone to inducing cytokine storms and autoimmune responses (71).

### 3.3 Effect of microbial community

The gut microbial community can influence the systemic immune system through various pathways, including the regulation of TLSs. The gut microbiota can regulate the immune function of gut-associated lymphoid tissue, which is interconnected with the systemic immune system and may influence the formation and function of TLSs through lymphocyte migration and circulation (72). At sites of tumors or inflammation, the local microbial community may also impact TLS effects. Some commensal microorganisms can influence the recruitment and activation of immune cells by interacting with them and modulating the secretion of local cytokines and chemokines (73). Furthermore, the composition and dynamics of microbial communities may affect local immune cell composition and function, such as altering the ratio of B cells to T cells and regulating macrophage polarization, which in turn influences the immune effects of TLSs (74, 75).

In a study on Epstein-Barr Virus-associated gastric cancer (EBVaGC), it was found that TLSs can serve as an independent prognostic factor for Epstein-Barr virus-negative gastric cancer (EBVnGC), but not for EBVaGC. In addition, the study found that the colonization of *Helicobacter hepaticus* has been shown to expand the colonic lymphatic network and promote mTLS formation, with evidence of H*. hepaticus* and *H. hepaticus*-specific T cells present in TLSs. Studies in mice and humans have indicated that TLSs and specific T cells can better control the development of colorectal cancer (76). So far, no research has shown that bacteria-related microorganisms in gastric cancer are associated with TLSs. Perhaps further exploration could be carried out on the microbiota of gastric cancer to clarify the possible connections and potential impacts between them and TLSs.

With the deepening understanding of the relationship between the gut microbiota and the immune system, microbiome-based therapies have gradually attracted attention. However, there are numerous potential risks associated with such therapies. In some cases, pathogenic infections or imbalances in microbial communities can lead to intensified inflammatory responses and immune dysfunction, negatively affecting TLS function and promoting tumor development or exacerbating inflammatory diseases (77). If microbiome-based therapies are not properly regulated, they may promote immune tolerance in the TME, help tumor cells evade the surveillance and killing of the immune system, which is unfavorable for the treatment and disease control of gastric cancer. In conclusion, the impact of the gut microbiota and its metabolites on TLSs is complex and multifaceted. When exploring microbiome-based therapies for the treatment of diseases such as gastric cancer, it is necessary to fully recognize the potential risks therein in order to achieve the goals of safe and effective disease treatment and immune regulation.

## 4 Effect of TLS-related B cells on immune response and clinical prognosis of gastric cancer

Increasing numbers of studies are demonstrating that B cells hold significant value in predicting the efficacy of immunotherapy for gastric cancer. TLS-related B cells within tumors exhibit different roles in the TME and immunotherapy, indicating that the role of B cells in this environment is complex and multifaceted (**Table 3**). A population of B cells characterized by higher activity and functionality may suggest a better therapeutic effect, potentially because these B cells can enhance the anti-tumor immune response of immunotherapy through multiple pathways, working in synergy with ICIs and other treatment methods to inhibit the growth and survival of gastric cancer cells. However, the specific mechanisms by which B cells operate in gastric cancer immunotherapy require further in-depth research.

**Table 3:** Multiple roles and mechanisms of B cells in tumors.

Functions	Mechanisms	Tumor type	Role in tumor	Refer
Anti-tumor	Mediates ADCP and ADCC actions by secreting anti-TAA antibodies.	Esophageal squamous cell carcinoma	ADCs produce IgG labeled tumor cells and play an IgG dependent anti-tumor role.	(210)
		Pancreatic cancer	Complement activation, Fc receptor binding on the surface of effector cells and ADCC involvement.	(16)
		Gastric cancer	MALT-B cells are involved in complement activation, immune response and B cell activation	(83)
	Through antigen driven clonal amplification, generic transformation and affinity maturation, change antigen targets and enhance the role of T cells.	Lung adenocarcinoma	Tumor-specific B cells interact with CD4+ T cells and Tfh cells to enhance CD8+ T cell action.	(211)
		Esophageal squamous cell carcinoma	NBC-NF-κ B cells highly express NF-κB related genes and unique genes encoding T cell stimulating proteins to enhance T cell activation.	(210)
		High grade serous ovarian cancer	PCs produce IgA to wrap the surface of tumor cells, which is recognized by immune cells and coordinates the reaction between tumor and T cells and B cells.	(212)
		Triple negative breast cancer	CD20+CD27+IgD subtype transformed B cells up regulate the expression of BCR pathway molecules and RGS1, and activate CD69 and TNF-α signal transduction.	(213)
	By promoting the recovery of tumor cells, leading to antigen uptake by antigen cell receptors and enhancing antigen presentation.	Pancreatic cancer	NACT-activated T cells stimulate CD40 molecules on B cells, promoting B cell activation through the NF-κB pathway and antigen presentation.	(214)
		Colorectal cancer	mIgG1/BCR mutant B cells lead to antigen uptake by Fcγ receptors on DCs and enhance DCs presentation.	(53)
	Activate CDC reaction by secreting related factors.	Oropharyngeal squamous cell carcinoma	B cells can produce chemokine CXCL9 and lymphotoxin, and recruit T cells to tumor tissues.	(215)
		Breast cancer	Complement regulatory protein CD55 regulates the production of icosl+B cell populations.	(80)
Pro-tumor	By producing immunosuppressive molecules, activate complement cascade reaction, differentiate into bregs, and inhibit cell function.	Breast cancer	TGF-β converts naïve CD4+ T cells into Tregs, limits effector T cell proliferation and function, and enhances Foxp3 and CTLA-4 expression.	(216)
		Pancreatic cancer	Inhibition of CD8+ T cell infiltration and promotion of IL-35 production by down regulating the secretion of CXCR3, CCR5 and IFN-γ.	(60)
		Melanoma	Differentiates TAMs to M2 phenotype and suppresses effector T cells and NK cells.	(217)
		Cervical cancer	Leads to degradation of the TCR ζ chain, resulting in a diminished T cell response.	(218)
		Liver cancer	Reduce the level of TNF-α through the production of IL-40 and TGF-β.	(219)
		Gastric cancer	IL-10 inhibits the production of IFN-γ and TNF-α in CD4+T cells, and TGF-β induces the proliferation of Tregs cells.	(98)

TAA, tumor-associated antigen; ADCP, antibody-dependent cellular phagocytosis; ADCC, antibody dependent cell-mediated cytotoxicity; ASCs, antibody-secreting cell; CDC, complement-dependent cytotoxicity; TAMs, tumor-associated macrophages; BCR, B cell receptor; TCR, T cell receptor.

**Figure 2:**
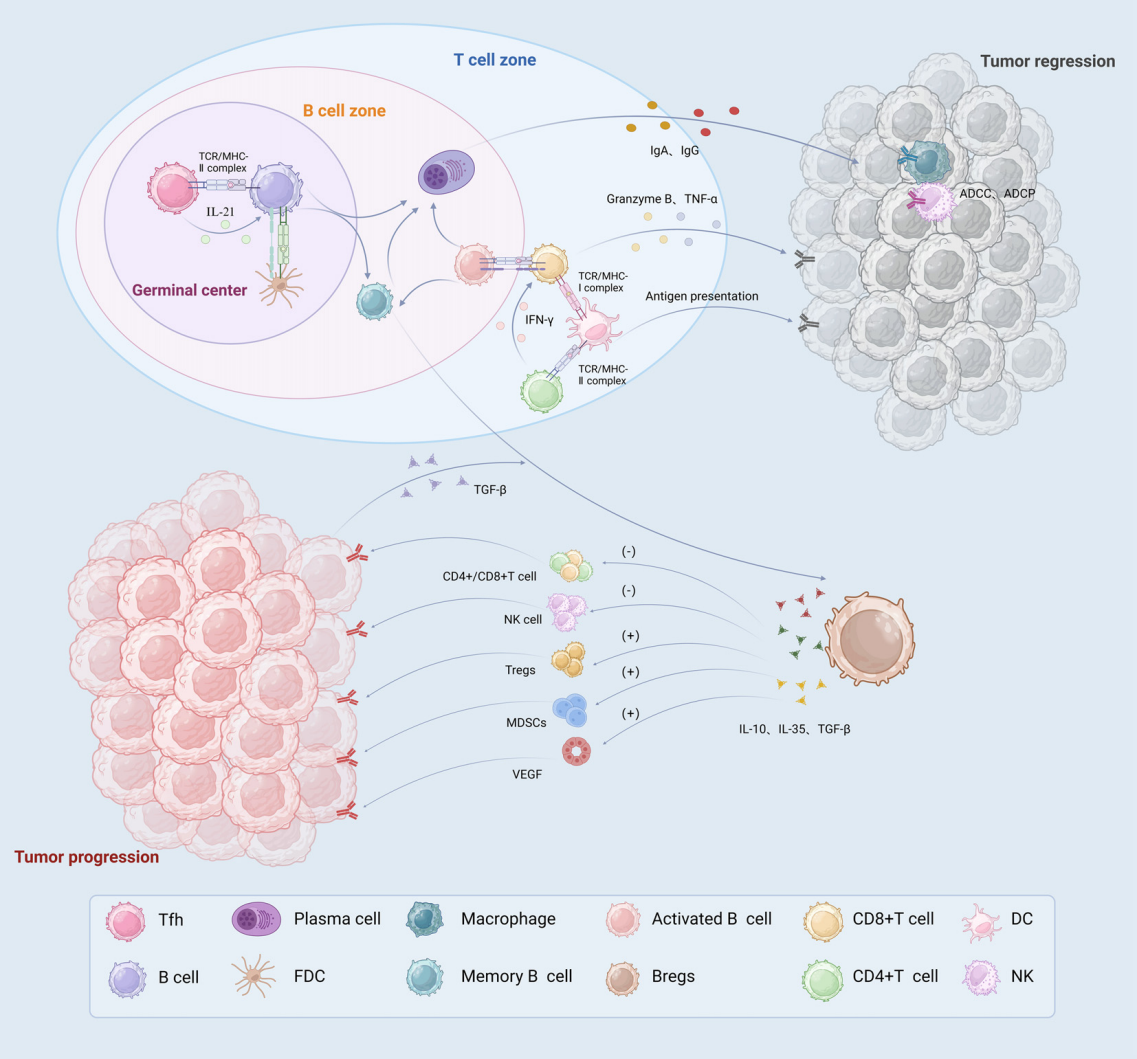
The immune role played by TLSs associated B cells in TME. In germinal centers, B cells and Tfh cells interact directly or indirectly to promote the differentiation of B cells into memory B cells and high affinity PCs. In the B cell zone, activated B cells not only act as presenting antigens, but also activate T cells to directly kill tumor cells. PCs produce tumor specific antibodies, such as IgA and IgG, which migrate along the fibroblast track to the tumor. They play an anti-tumor immune role through the ADCC and ADCP effects mediated by macrophages and NK cells. DCs located in the T cell zone exert antigen-presenting effects and activate T cells. CD8+T cells directly recognize specific antigens and kill tumor cells by releasing TNF-α and granzyme B CD4+T cells secrete IFN-γ and help activate CD8+T cells. Bregs release IL-10, IL-35 and TGF-β immunosuppressive factors, which promote tumor growth by inhibiting T cell and NK cell effector functions and promoting Tregs, MDSCs and angiogenesis. PCs, plasma cells; ADCP, antibody-dependent cellular phagocytosis; ADCC, antibody dependent cell-mediated cytotoxicity; Bregs, regulation B Cell; Tregs, regulatory T cells.

The following sections will outline the roles of TLS-associated B-cell subsets in the gastric cancer immune microenvironment, providing new possibilities for targeting gastric cancer immunotherapy (**Figure 2**).

### 4.1 Antibody production

The anti-tumor properties of B cells are primarily mediated by PCs that produce antibodies, which are associated with high survival rates in various solid tumors (78). Within the germinal centers of mTLSs, disseminated along CXCL12-containing fibroblast pathways, B cells differentiate after undergoing antibody class switching and somatic hypermutation to produce Ig antibodies targeting tumor-associated antigens, correlating with improved immunotherapy responses (79). It has been found that overall survival (OS), effectiveness, and clinical benefit of anti-programmed death receptor-1 (anti-PD-1) therapy are significantly higher with higher scores, particularly for the IgA1, IgA2, IgG1, and IgG2 isotypes (80).

TLSs and B cells serve as important predictors of patient prognosis; an increase in TLSs and CD20+ B cells leads to a corresponding increase in PC density, resulting in the production of specific tumor-fighting antibodies (81, 82). Studies have shown that a significant number of MALT-B cells have been detected in patients who have gastric adenocarcinoma with mTLSs. The function of these MALT-B cells was linked to complement activation, immune response, and B-cell activation pathways. Moreover, IgA was highly expressed in MALT-B cells and was secreted directly to nearby mucous membranes upon antigenic stimulation, indicating that IgA-mediated humoral immunity plays an important role in promoting anti-tumor immunity in patients with gastric cancer (83).

Additionally, the proportion of IgG PCs around TLSs was found to be significantly higher in both normal and tumor tissues, while no significant change was observed in IgA PCs. It is hypothesized that TLSs may serve as the central locus for IgG PC production, with CD20+ B cells located within TLSs differentiating into IgG PCs, producing antibodies to enhance the anti-tumor immune response (67). The B-cell population is mainly divided into two types: CD20+ B cells and CD138+ PCs. CD20+ B cells are predominantly found in TLSs, and their proportion in gastric cancer tissues is approximately three times that in adjacent tissues. Correlation analysis shows that the density of CD20+ B cells is positively correlated with the density of T cells, suggesting a synergistic role of B cells and T cells in anti-tumor immunity (84).

Because of the presence of a large amount of sulfated glycosaminoglycans, CD20+ B cells also act as a protective factor independent of other prognostic factors in gastric cancer patients. These cells are the main functional B-cell antigens in gastric cancer, and their production of anti-sulfated glycosaminoglycan antibodies shows significant tumor growth inhibition (84, 85).

### 4.2 Antigen presentation

Memory B cells in TLSs can induce the expansion of related T cells and transform into PCs to participate in anti-tumor immunity. As a special type of APCs, these cells are distributed in the germinal center and B-cell regions of TLSs, where they assist in B-cell activation and antibody production (12). A study identified an antigen-presenting B cell within the lymphoid follicles of TLSs, capable of inducing T-cell responses and phenotype transitions (86). In studies of Epstein–Barr virus-related gastric cancer samples, the expression of MHC-II mRNA was significantly increased, highlighting the important role of tumor-associated cells in antigen presentation (87).

Tumor-associated macrophages (TAMs) are present in primary follicle-like TLSs (88). TAMs also play a major antigen-presenting role and exist in two subtypes: classically activated M1 and alternatively activated M2, which can interconvert. The relationship between TAMs and TLSs in cancer is an issue worthy of exploration. Due to the complexity of TAMs in the TME, different subtypes of TAMs located in TLSs also play different roles in the prognosis and immune responses of cancer patients. In patients with gastric cancer, expression levels of CD68+ TAMs and TLSs are negatively correlated; high expression of CD68+ TAMs usually indicates low expression of TLSs, suggesting an association between TAM presence, gastric cancer progression, and lower survival rates (89, 90). Studies have shown that M2-polarized TAMs are linked to poor prognosis in gastric cancer, as they inhibit T-cell anti-tumor responses through regulatory factor secretion, leading to immune evasion by gastric cancer cells (91). For example, IL-10 expressed by TAMs can inhibit antigen presentation by downregulating MHC molecule expression in tumor cells. TAMs expressing IL-10 are associated with CD8+ T-cell dysfunction, as well as features of gastric cancer such as the Epstein–Barr virus status, programmed death ligand-1 (PD-L1) expression, and genomic stability, underscoring the importance of TAMs as potential prognostic biomarkers for gastric cancer and their role in promoting immune evasion through IL-10 production (92).

### 4.3 Immune response regulation

Although numerous studies have explored the composition of immune cell subsets within TLSs in gastric cancer, none have directly conducted detailed detection and analysis specifically for regulatory B cells (Bregs). Consequently, it remains uncertain whether Bregs are present in TLSs. However, certain research has indicated that there are B cells within TLSs that secrete immunosuppressive factors, a characteristic similar to that of Bregs. Given that B cells are a crucial component of TLSs, it is possible that Bregs, as a subset of B cells, may also be involved. Nevertheless, further confirmation is required, and this could potentially become a focus of future research.

Bregs can release negative regulators such as IL-10, IL-35, and TGF-β to inhibit the immune response, suppress the activity of T cells and natural killer cells, and induce Tregs, myeloid-derived suppressor cells (MDSCs), and angiogenesis, facilitating tumor immune escape (93). IL-10 has a dual function, capable of both promoting and inhibiting tumor growth (94). Bregs inhibit Th1/Th17 cell differentiation by producing IL-10, thereby aiding tumors in immune evasion (95). Bregs induced by tumor cells can also suppress anti-tumor responses and upregulate Tregs, promoting cancer metastasis (96). The proliferation-inducing ligand (APRIL) can promote the production of IL-10 by IgA+ B cells exhibiting a Bregs phenotype (97). CD19+CD24^hi^CD38^hi^ cells in gastric cancer are the primary source of IL-10 and can significantly inhibit the secretion of IFN-γ and TNF-α by CD4+ T cells (98).

When analyzing the prognostic impact of TAM subgroups expressing IL-10 in patients with gastric cancer, it was found that those with high IL-10 expression had a poorer prognosis and reduced responsiveness to adoptive cell therapy. Only patients with low infiltration of IL-10-expressing TAMs showed favorable outcomes with adjuvant chemotherapy (92). IL-10 influences the ability of tumor cells to proliferate, invade, and metastasize by inhibiting apoptotic signaling pathways and modulating cytokine production and release. For example, IL-10 expressed by TAMs was significantly elevated in the tissues and sera of patients with gastric cancer, inducing immunosuppression by regulating the c-Met/STAT3 signaling pathway. c-Met serves as an upstream regulator of STAT3, and its upregulation enhances the expression and phosphorylation of STAT3, which, when activated, can participate in the regulation of cancer proliferation and metastasis. This indicates that STAT3 indirectly affects IL-10’s influence on the proliferation, invasion, and migration of gastric cancer cells (99). Additionally, the anti-inflammatory lipid mediator lipoxin A4 inhibits Bregs through STAT3 and ERK dephosphorylation, thereby limiting tumor cell growth (100).

IL-35 converts B cells and T cells into IL-10 and IL-35 producing Bregs and Tregs, promoting tumor growth and metastasis (101). Studies have shown that the immunity of IL-35-deficient mice is enhanced; not only are macrophages and T cells activated, but the antigen-presenting ability of B cells is also significantly improved (102). Research indicates that IL-35-producing B cells exert their effects through STAT3 phosphorylation, and when STAT3 phosphorylation is inhibited, autocrine IL-35 does not impact B-cell expression. However, these cells can still self-expand through STAT3 phosphorylation (103).

The significant role of IL-35 in various tumors, including hepatocellular carcinoma (104), advanced breast cancer (105), and prostate cancer (106), has been well established. Recent studies have found that B cells in patients with gastric cancer can produce IL-35, inducing immunosuppressive effects on tumors. The frequency of IL-35 production correlates with Treg cells, MDSCs, IL-10-producing B cells, and CD14 monocytes, indicating that the inhibitory effect of IL-35 requires interaction with other immunosuppressive cells. This interaction may serve as a potential prognostic biomarker for patients with gastric cancer (107).

At the early stage of cancer development, TGF-β inhibits cancer by regulating target genes, inhibiting cell cycle protein-dependent kinases, and blocking the cell cycle’s developmental phase (62). Kinker et al. (108) found that reactive T cells exposed to TGF-β produce CXCL13, which in turn induces the formation of TLSs. TGF-β is associated with the development of gastric cancer, with TGF-β1 being the most commonly expressed form. TGF-β1 promotes metastasis by penetrating the basement membrane and enhancing the adhesion activity of gastric cancer cells (109). TGF-β not only induces Treg differentiation but also promotes the production of TGF-β-secreting CD4+ T cells and IL-10-secreting Tregs. In gastric cancer, CD19+CD24^hi^CD38^hi^ Bregs can produce TGF-β, and studies have confirmed that the conversion of CD4+CD25− effector T cells to CD4+FoxP3+ Tregs by CD24^hi^CD38^hi^ cells is dependent on TGF-β1 (98).

It has been demonstrated that low mRNA expression of TGF-β1 is associated with prolonged OS in patients with gastric cancer. Knockdown of TGF-β1 and its receptors in gastric cancer cells has been shown to block cell proliferation, migration, and invasion, indicating that TGF-β could be further explored as a therapeutic target (110). However, in later stages of tumorigenesis, TGF-β contributes to genetic instability, induces EMT, and promotes neoangiogenesis, facilitating tumor cell invasion and metastasis (111, 112).

When analyzing gene expression in gastric cancer cell lines, researchers found that two gene groups with significant expression differences were related to EMT. Given the prominent role of TGF-β signaling in EMT, it was observed that the expression of SMAD3/7 and TGF-β was significantly higher in the EMT group. Further investigation revealed that TGF-β had an inhibitory effect in cell lines of the non-EMT group but did not affect cell growth in the EMT group (113). Previous studies have shown that gastric cancer increases Treg levels through the TGF-β signaling pathway under hypoxic conditions, and high expression of hypoxia-inducible factor-1α (HIF-1α) promotes EMT. Therefore, TGF-β-mediated EMT can be inhibited by interfering with HIF-1α. Further research has identified that dextran sulfate targets HIF-1α, inhibiting EMT in gastric cancer cells at both mRNA and protein levels, suggesting that HIF-1α and dextran sulfate hold promise for gastric cancer immunotherapy (114).

Additionally, MiRNAs also regulate EMT in gastric cancer cells. For example, MiR-381 is significantly reduced in gastric cancer cell lines and can inhibit the TGF-β signaling pathway, suppressing EMT expression by targeting transmembrane protein 16A (115).

### 4.4 Biomarkers for predicting the prognosis of gastric cancer

The presence of PCs in TLSs is associated with enhanced anti-tumor immune responses and a favorable prognosis in cancer. As a subtype of B cells, PCs play a positive prognostic role in tumorous gastric mucosa. Mott cells, which are PCs containing Russell bodies, have been associated with higher survival rates in patients with gastric cancer. Studies suggest that the presence of Mott cells in gastric cancer tissue correlates with better patient outcomes than in cases where these cells are absent (116). During the histological analysis of gastric cancer tissues, certain plasma cell-like cells displaying the features typical of Mott cells were noticed in the areas of tumor-associated TLSs. Nevertheless, at present, additional studies are needed to confirm that these cells are truly Mott cells. It is possible that this finding might open up a new avenue as a potential target for the treatment of gastric cancer. Liu et al. identified a novel immunosuppressive molecule, the T-cell immune receptor (TIGIT), which is highly expressed on CD20+ B cells and is linked to poorer OS and disease-free survival (DFS) in patients with gastric cancer by influencing CD8+ T-cell depletion (12).

In renal clear cell carcinoma, it was observed that tumor cells labeled with IgG exhibited better progression-free survival (PFS) (117). Italiana et al. also found that in TLSs of soft tissue sarcoma, PCs expressing IgG were significantly associated with improved immune response and prognosis (118). Regarding gastric cancer progression and prognosis, Okita et al. discovered that tumor-infiltrating CD11b+ cells induced Treg formation in the gastric cancer microenvironment, showing a strong correlation between high expression of CD11b APCs and poor prognosis in patients with gastric cancer (119).

CD208 is a biomarker for mature DCs, and studies have shown that CD208 cell infiltration is linked to poor prognosis in colon cancer (120, 121). Given that most gastric cancer tissues are adenocarcinomas, similar to colon cancers, Ishigami et al. demonstrated that CD208 cell infiltration in gastric cancer was also negatively correlated with patient prognosis (122). Additionally, IL-10 released by Bregs at hypomethylation has been associated with poor prognosis and worse OS in patients with gastric cancer (123).

Cottrell et al. observed abundant PCs and TLSs in tumor specimens excised following PD-L1 treatment, both of which are key features of immune-mediated tumor regression (124). Jiang et al. conducted an integrated analysis of TLS scores in patients with gastric cancer, finding that those with high TLS scores had higher survival rates (125).

## 5 Role of TLSs in clinical immunotherapy of gastric cancer

### 5.1 Role of TLS-related chemokines in gastric cancer

CXCL13, also known as B lymphocyte chemokine, is primarily derived from Tfh cells but can also be secreted by tumor-associated cells, such as macrophages, DCs, and specific CD8+ T cells (126, 127). CXCL13 plays a crucial role in maintaining the structure of TLSs, promoting germinal center responses, and supporting humoral immunity, particularly by promoting the production and recruitment of B cells within TLSs (67). In recent years, CXCL13 has been applied as an alternative marker for TLSs in pathological situations. Henry et al. found that CXCL13 is present in lymphocyte infiltration, driving B lymphocyte recruitment to inflamed islets. They also discovered that blocking CXCL13 disrupts the histomorphology of B lymphocytes in TLSs, although it does not significantly alter B lymphocyte proportions (128).

Using TLSs as a focal point, Hu et al. uncovered key roles for TLS-associated B cells and CXCL13-expressing CD103+CD8+ tissue-resident memory T cells (Trm) in anti-tumor immunity. They found that activated B cells could stimulate the mTOR signaling pathway in CD103+CD8+ Trm cells through the LTα-TNFR2 signaling axis, promoting glycolysis and the secretion of CXCL13 and granzyme B, which in turn kills gastric cancer cells (129). CXCL13 selectively binds to the chemokine receptor CXCR5, and their interaction is essential for maintaining B-cell homeostasis and activation (65, 130). CXCR5 is expressed significantly in B cells, Tfh cells, mature DCs, and tumor cells (127, 131).

Differential expression analysis of CCR5 in gastric cancer tissue through immunostaining revealed that high expression of CCR5 is associated with lymph node metastasis and poor prognosis in patients with gastric cancer (132). Additionally, a study of cellular content in gastric cancer tissue showed that CXCR5+CD8+ T cells are predominantly present in both gastric cancer and paracancerous tissues. Patients with gastric cancer who exhibited high infiltration of CXCR5+CD8+ T cells had longer OS than those with lower infiltration of CD8+ T cells (133).

Multiple cytokines, intercellular interactions, and signaling pathways regulate the CXCR5–CXCL13 axis, and variations in their expression and regulation can impact the characteristics of the tumor immune microenvironment and immune cell functions. For instance, CXCL13 and CXCR5 co-expression in cancer not only promotes proliferation and migration but also serves as a prognostic marker due to its activation of the PI3K/AKT/mTOR pathway via CXCR5 (134). CD40, a cell surface co-stimulatory molecule, induces immune escape by inhibiting T-cell expansion and recruiting MDSCs (135). Research has shown that CD40 can regulate the expression of CXCR5 in MDSCs, influencing their recruitment and accumulation in gastric cancer. Therefore, targeting CD40 or the CXCR5-CXCL13 axis could be a promising strategy for treating gastric cancer (136).

CCL19 is expressed by DCs and cancer cells and plays a critical role in T-cell response and anti-tumor immunity, making it a potential prognostic and immunotherapy biomarker as well as a target for cancer treatment (137–139). CCL21, a secondary lymphoid tissue chemokine, is expressed by HEVs, lymphatic endothelial cells, and cancer cells. It binds to glycosaminoglycans, anchoring to the surface of endothelial cells (138, 140). Both CCL19 and CCL21 are expressed in lymph nodes, with the distinction that CCL21 is produced in the T-cell zone and then translocated to HEVs (68, 69). CCL21 has been found to be overexpressed in patients with gastric cancer, and further studies have shown that it upregulates the expression of MALAT1, activates the mTOR signaling pathway, and promotes migration, invasion, and epithelial–mesenchymal transition (EMT) of gastric cancer cells (141).

CCR7 is involved in various biological processes such as lymph node homeostasis, immune tolerance, inflammatory response, and cancer metastasis. It primarily recognizes CCL19 and CCL21 produced by T cells in SLOs and facilitates their migration through HEVs (66, 142, 143). In database analyses of chemokines in gastric cancer samples, the expression of CCL19 and CCR7 was significantly higher in gastric cancer samples compared with normal tissues. Patients with high expression levels exhibited poorer OS, suggesting that CCL19/CCR7 can serve as indicators of poor prognosis in patients with gastric cancer (144).

Zhou et al. demonstrated that CCL19 enhanced the activity of AIM2 via CCR7, significantly inhibiting the invasion and migration of gastric cancer cells (145). CCR7 is expressed in gastric epithelial cells of non-inflamed gastric mucosa, *H. pylori* gastritis, intestinal epithelial chemotaxis, ectopic hyperplasia, and gastric cancer. It is upregulated by *H. pylori*, but not by cytotoxin-associated genes (146). A research team explored the effect of CCR7 on TGF-β1-induced EMT for the first time, showing that CCR7 acts as a co-stimulator of TGF-β1 in gastric cancer, with NF-κB signaling also playing a role in this process (147).

Research indicates that TNF-α derived from gastric cancer induces the production of CD45RA−CCR7+ Treg subsets by activating STAT3 phosphorylation. Through IL-10 secretion and cellular contact mechanisms, CD45RA−CCR7+ Tregs exhibit strong inhibitory effects on CD8+ T cells, thereby promoting the growth of gastric cancer (148).

### 5.2 Prognostic factors of TLSs in gastric cancer

The TME is a complex environment composed of various subgroups of immune cells, playing a significant role in tumor development, progression, and treatment outcomes. Studies have shown that the presence of TLSs is associated with the survival and treatment response of patients with cancer. The density, location, and maturity of TLSs within tumors, as well as the presence of HEVs, may influence patient prognosis. Different studies have reported the impact of TLS distribution, density, cell composition, and maturity stages on patient outcomes (**Table 4**).

**Table 4:** Relationship between multiple factors related to TLSs and tumor prognosis.

Factors	Tumor type	Relationship with prognosis	Refer
Density	Right sided colon cancer	TLSs density is positively correlated with the prognosis of patients with right sided colon cancer.	(220)
	Gastric cancer	The prognosis of patients with high TLSs density is better than that of patients with low TLSs density; After treatment with navulizumab, patients with TLSs density had longer PFS.	(221)
	Lung squamous cell carcinoma	The high density of TLSs is related to the improvement of PFS, DFS and OS.	(14)
	Hepatocellular carcinoma	TLSs density was positively correlated with survival and NLR was positively correlated with OS in HCC patients, and the combination of TLSs density and NLR better predicted survival in patients.	(155)
	Esophageal cancer	The high density of TLSs was positively correlated with NLR and survival time.	(156)
Location	Breast cancer	The presence of peritumoral TLSs is associated with poor DFS and OS.	(151)
	Hepatocellular carcinoma	High density intratumoral TLSs are associated with low recurrence rate, while para cancerous TLSs have no prognostic value.	(152)
	Intrahepatic cholangiocarcinoma	High density intratumoral TLSs indicate good prognosis, while the presence of peritumoral TLSs is associated with poor prognosis.	(153)
	Renal clear cell carcinoma	The presence of proximal TLSs was significantly associated with the improvement of PFS and OS, while the presence of distal TLSs was associated with poor PFS and OS.	(154)
	Head and neck squamous cell carcinoma	The proximity of TLSs to tumor cells was positively correlated with prognosis.	(150)
Maturity	Esophageal cancer	Compared with patients with less mTLSs, patients with abundant mTLSs have better prognosis.	(192)
	Lung squamous cell carcinoma	Neoadjuvant chemotherapy damages the germinal center, affects maturation of TLSs and diminishes their prognostic capacity.	(14)
	Non-small cell lung cancer	NSCLC patients with high TLSs maturity showed better DFS.	(160)
	Laryngeal squamous cell carcinoma	High maturity TLSs are independent positive prognostic indicators in laryngeal carcinoma.	(161)
HEVs	Intrahepatic cholangiocarcinoma	Patients with high levels of HEVs have better OS.	(162)
	Colorectal cancer	Patients with high levels of HEVs are associated with longer OS, DFS and lower TNM staging.	(163)
	Non-small cell lung cancer	The low expression of ICLs in mature HEVs was positively correlated with better DFS.	(164)
	Gastric cancer	Patients with high levels of CD8+/HEVs showed the longest overall survival.	(165)

PFS, progression survival; DFS, disease free survival; OS, overall survival; NLR, neutrophil to lymphocyte ratio; ICLs, immune checkpoint ligand.

#### 5.2.1 TLS location and heterogeneity

TLSs in cancer can be classified as peritumoral (located around the tumor) or intratumoral (located within the tumor). The prognosis of patients with different tumors may vary depending on the location of TLSs. This variability is partly due to TLS heterogeneity, which suggests that in some cases, TLSs may act as negative prognostic factors (32, 149). For instance, in patients with head and neck squamous cell carcinoma, the proximity of TLSs to tumor cells serves as a key indicator of response to immunotherapy, with patients whose TLSs are distant from tumor cells showing poorer outcomes (150). In breast cancer, the presence of peritumoral TLSs is associated with poor DFS and OS (151). In hepatocellular carcinoma, high-density intratumoral TLSs are linked to lower recurrence rates, whereas TLSs located in the paracancerous area are not significantly associated with prognosis (152). Similarly, in patients with intrahepatic cholangiocarcinoma, high-density intratumoral TLSs are associated with a better prognosis, while peritumoral TLSs correlate with poorer outcomes (153). However, the findings in renal clear cell carcinoma contradict this pattern, indicating that the impact of TLSs on the prognosis may depend on their location and composition (154).

When TLSs are situated within the tumor mass itself, they can directly impact the local immune microenvironment within the cancerous tissue, a more active immune response can be initiated precisely at the core of the tumor. While TLSs located in the peritumoral area, may play a role in either containing tumor spread or facilitating the interaction between the tumor and the systemic immune system. In clinical trials, gastric cancer patients can be stratified for treatment based on the distinct locations of TLSs within the tumor. Patients with intratumoral TLSs are more likely to exhibit a favorable response to immune checkpoint inhibitors and similar immunotherapeutic approaches. For peritumoral TLSs, the immunosuppressive factors around the tumor should be addressed first before initiating standard immunotherapy. This approach aims to optimize the treatment outcome by tailoring the therapeutic strategy according to the specific characteristics associated with the location of TLSs in each patient.

#### 5.2.2 TLS density

The density of mTLSs containing germinal centers is generally indicative of a favorable prognosis, as demonstrated in various solid tumors. In hepatocellular carcinoma, TLS density alone is positively correlated with patient survival, and a combined analysis with the neutrophil-to-lymphocyte ratio provides a better prediction of patient survival rates (155). In lung squamous cell carcinoma, higher TLS density is associated with significant improvements in PFS, DFS, and OS (14). Similarly, high-density TLSs in esophageal cancer have been shown to significantly improve NLR and survival rates (156).

In gastric cancer, the B-cell zone of TLSs is primarily composed of CD20+ B cells. The expression of CD20+ B cells in high-density TLS tissues is significantly greater than in low-density TLS tissues, and high infiltration levels of CD20+ B cells are associated with lower lymph node metastasis, lower TNM staging, and longer OS and DFS (89, 157). Hennequin et al. observed a significant correlation between the density of B-cell aggregates in gastric cancer and Tbet+ effector T cells, which is linked to higher recurrence-free survival rates. This finding suggests that gastric cancer cells can be stabilized through the coordination of tumor-infiltrating T cells and B cells within TLSs (158).

It has been discovered that TLSs with high density tend to display a more robust immune response directed towards tumors. This is likely attributable to the fact that immune cells within these TLSs play a significant anti-tumor immune role. In the context of future clinical therapies, it could be taken into account that patients with a high density of TLSs be subjected to treatments of relatively lower intensity. Conversely, patients with low TLSs density might have a weaker immune response. These patients could be targeted with more aggressive treatment combinations, such as combining chemotherapy with immunotherapy or using experimental agents that aim to increase TLSs density.

#### 5.2.3 TLS maturity

mTLSs are defined as aggregates of immune cells that include germinal centers. Studies have shown that the presence of mTLSs is associated with an improved objective response rate, PFS, and OS (159). In lung squamous cell carcinoma, the proportion of TLSs containing germinal centers was found to decrease in patients receiving neoadjuvant chemotherapy, suggesting that TLS maturation was compromised and impacting patient prognosis (14). In patients with non-small cell lung cancer, the maturity of TLSs is closely linked to the effectiveness of neoadjuvant therapy, and it serves as an independent predictor of DFS (160). In laryngeal squamous cell carcinoma, high-maturity TLSs have been observed to contain richer immune cell infiltration and a more robust immune response, whereas low-maturity TLSs are associated with tumor invasion, metastasis, and immunosuppressive pathways (161).

Mature TLSs have been demonstrated to be correlated with a more efficacious immune response. The germinal centers present within mTLSs are capable of facilitating processes like the maturation of B cells and the production of antibodies. In the realm of clinical treatment, gastric cancer patients can be stratified based on the maturity level of TLSs. For patients whose TLSs are mature, standard immunotherapy can be adopted. However, for those with immature TLSs, additional treatments might be necessary to foster the maturation of TLSs, such as the application of specific cytokines, with the aim of attaining a more favorable response to subsequent immunotherapy or other anti-tumor therapies.

#### 5.2.4 HEVs

Studies have shown that HEVs can be key prognostic factors in various tumors. In hepatocellular carcinoma, a high density of HEVs significantly improves patient prognosis and may serve as an independent prognostic indicator (162). In colorectal cancer, analysis of the HEV/TLS ratio revealed that patients with a high HEV/TLS ratio had longer OS and DFS, as well as increased recruitment of T cells and macrophages, suggesting that a high HEV/TLS ratio is associated with a favorable prognosis (163).

A scoring system was developed by detecting HEV and immune checkpoint ligand (ICL) expression in lung adenocarcinoma tissues. It was found that ICL expression on HEVs can predict patient survival, with high ICL scores indicating a poorer prognosis (164). HEVs have also been identified as alternative biomarkers for T-cell inflammation in the TME and as prognostic indicators in gastric cancer. Researchers found a favorable prognostic role for HEVs in gastric cancer by analyzing the combination of CD8+ and Foxp3+ TILs with HEVs (165, 166).

HEVs are essential for facilitating the entry of immune cells into the TLSs and the tumor microenvironment. In clinical trials, patients can be stratified based on HEVs expression levels. Those with high HEV expression might be enrolled in trials with less intensive treatment, while those with low HEV expression could be targeted with more aggressive approaches. In addition, changes in the expression of HEVs can also be monitored to make accurate judgments regarding the intensity of subsequent treatments.

### 5.3 Predicting the effect of immunotherapy

An increasing amount of data suggests that cancer therapies stimulating anti-tumor immune responses are more effective in generating long-term outcomes. TLSs, a key component of the tumor immune environment, are the primary sites for generating anti-tumor immune responses, and their presence, along with B-cell infiltration, can help predict patient responses to immune checkpoint blockade (ICB) (167). ICB enhances the immune system’s ability to attack tumor cells by inhibiting immune checkpoint molecules, such as PD-1, PD-L1, and CTL-associated protein-4 (CTLA-4), and by reducing T-cell inhibition within the TME (168).

There is an association between TLSs and ICB, where TLSs predict ICB treatment efficacy and ICB infers TLS density and status. In patient samples treated with ICB neoadjuvant therapy, it was observed that CD20+ B-cell density, TLS density, and the TLS-to-tumor area ratio increased significantly, indicating that B cells in TLSs contribute to the response of patients to ICB (167). Research has shown that the presence of mTLSs can serve as a predictive factor for immune therapy response in patients receiving ICI treatment. Regardless of PD-L1 expression, mTLS status is associated with better efficacy outcomes and improved survival rates (159).

A retrospective analysis of patients with gastric cancer and other tumors revealed that mTLSs present before ICI treatment were associated with better outcomes, and TLS composition was found to be a more reliable predictor than TLS density (11).

### 5.4 Application of TLSs in clinical immunotherapy of gastric cancer

Immunotherapy for tumors has increasingly become a prominent field in the treatment of solid tumors. TLSs, as unique structures of immune cells within the TME, have garnered significant attention. Treatment strategies involving the induction of TLSs are being applied in cancer therapy, including the use of TLS-related cytokines or chemokines, immunotherapy, cancer vaccines, chemotherapy, and radiotherapy (**Table 5**). These approaches can induce the formation of local TLSs and enhance the anti-tumor immune response, and they may promote the activation of autoreactive T and B cells (169).

**Table 5:** Treatment induction of TLSs.

Induction methods	Specific molecule	Refer
Cytokine induction	LIGHT	(171)
	STING	(172)
	TLR	(173)
	LTα and LTβ	(174)
	CXCL13	(175, 176)
	CCL5	(177)
	CXCL12	(178)
Immune cell induction	CAFs	(179)
	CD8+T	(180)
	DCs	(182)
	CX3CR1hi macrophages	(183)
Cancer vaccine induction	Antigen therapeutic vaccine	(184)
	Nanovaccine	(185)
	GVAX	(186)
	AS15 vaccine	(187)
Immunotherapy induction	Anti PD-1	(124, 167, 190, 191)
	Anti CTLA4	(190)
Chemotherapy induction	N.A.	(14, 80, 192, 193)
Radiotherapy induction	N.A.	(194)
Artificial induction	3D scaffolds	(195)
	Collagen sponge scaffolds	(196)
	STING activator hydrogel	(197)

#### 5.4.1 Cytokine induction

The use of cytokines, small molecule drugs, or biologics can promote the formation and maturation of TLSs (170). For example, TNF superfamily member TNFSF14 (LIGHT) acts as a ligand for LTβR and is a key molecule in TLS formation. LIGHT recruits large numbers of T cells into tumors to enhance immune efficacy and can also induce TLS formation by remodeling the tumor vasculature (171). In a melanoma mouse model, a low-dose injection of an IFN gene stimulating protein (STING) agonist promoted vascular normalization, induced TLS formation, and inhibited tumor growth (172).

Toll-like receptor agonists may affect the expression of CD19, CD20, and CXCL13 mRNA in the thymus by activating relevant signaling pathways, modulating immune cell behavior and influencing TLS formation (173). High expression of LTα and LTβ in colorectal cancer promotes TLS formation through the interaction between LTα1β2 and LTo cells (174). In mouse models of pancreatic and ovarian cancer, the injection of CXCL13 induced TLS formation, enhancing the synergistic anti-tumor response of cellular and humoral immunity (175, 176). Endogenous cyclic guanosine monophosphate synthase promotes endothelial cells to produce CCL5, recruiting CD8+ T cells to TLSs through CCR5 signaling, leading to TLS formation and improved anti-tumor immunity (177). CXCL12 and its receptor CXCR4 have also been shown to induce TLS formation in breast cancer (178).

#### 5.4.2 Immune cell induction

Chemokines attract specific immune cells to targeted sites, and by regulating chemokine expression, the local aggregation of immune cells within the tumor can be promoted, leading to TLS formation. For example, cancer-associated fibroblasts (CAFs), which are prominent in non-small cell lung cancer, can significantly increase CD4+ and CD8+ T cells through TGF-β, inducing the production of CXCL13 and promoting TLS formation (179). Studies have shown that CAFs in abdominal tumors can function as LTo cells, while CD8+ T cells and B cells can act as LTi cells, coordinating to drive the formation of tumor-associated TLSs (180).

Additionally, CAFs express CXCL13, B-cell activating factor, and APRIL. Subcutaneous injection of these CAFs has been shown to promote the formation of tumor-associated TLSs, which are inversely correlated with tumor growth. Following immune checkpoint therapy, the number of B cells significantly increases, inducing the formation of additional TLSs (180). In advanced gastric adenocarcinoma (gastric signet ring cell carcinoma), enhancing CD8+ T cells’ ability to produce CXCL13 can stimulate TLS formation in patients, exerting anti-tumor effects (181).

DCs expressing Tbet (DC.Tbet) produce the IL-36γ pro-inflammatory gene, and injecting these DCs into tumors has been shown to slow tumor growth and promote TLS formation, although the B cells within these TLSs do not form germinal centers (182). Another study identified CX3CR1hi macrophages in the intestinal mucosa as the primary cells driving TLS formation, capable of inducing IgA responses through antigen presentation within TLSs (183).

#### 5.4.3 Cancer vaccine induction

Targeting TLSs as vaccine sites can activate their immune functions by focusing on specific cells or molecules. For instance, in patients with cervical cancer, therapeutic vaccines increase the expression of CD8+ T cells in the lesion epithelium, leading to the formation of TLSs with germinal centers that produce a strong immune response (184). Utilizing cytokines or chemokines within TLSs as adjuvants can also enhance vaccine efficacy. A nanovaccine developed by Wen et al. increased the expression of CCL19, CCL21, CXCL10, and CXCL13 through activation of the LT-α and LT-β pathways, promoting TLS formation, enhancing the local immune response, and delaying tumor growth (185).

Introducing immunostimulatory genes into tumor or immune cells can further enhance immune cell activity and promote TLS formation. For example, administer solely an irradiated, granulocyte-macrophage colony-stimulating factor (GM-CSF)-secreting, allogeneic vaccine known as GVAX to patients suffering from pancreatic ductal adenocarcinoma (PDAC), allowing the vaccine antigen to be more effectively absorbed by immune cells within TLSs. After 2 weeks of treatment, TLSs were observed to be highly clustered, and patient survival periods were significantly extended (186). After the administration of an AS15 vaccine, which is synthesized from a TLR4 agonist, a TLR9 agonist, and QS-21, to patients suffering from melanoma, PNAd and blood vessels were detected in samples, indicating that the AS15 vaccine induces the formation of TLSs containing HEVs (187).

#### 5.4.4 Immunotherapy induction

Unlike previous treatment methods, tumor growth is currently inhibited primarily through the anti-tumor effects exerted by ICI when activated (188). Studies have shown that combined treatment with anti-CTLA-4 and anti-PD-1 increases the number of TLSs and B cells, leading to tumor regression (180, 189). In patients with urothelial carcinoma, combined treatment with PD-1 and CTLA-4 before tumor resection significantly increases TLS density within the tumor, indicating that TLSs are induced and recruited during treatment, activating B cells to initiate anti-tumor immune responses (190).

Cottrell et al. observed an abundance of PCs and TLSs in tumor specimens resected after PD-L1 treatment, similarly demonstrating that TLS formation is induced following PD-L1 therapy (124). In non-small cell lung cancer, anti-PD-1 therapy not only enhances B-cell activation but also induces tumors to produce CCL21 and increases the number of TLSs with antibody production, thereby boosting the immunotherapy response (191). Helmink et al. also found that ICI treatment promotes TLS formation, and patients with highly expressed TLSs are more likely to respond favorably to treatment (167). In a single-arm feasibility trial, the combination of CTLA-4 and PD-1 was observed to induce TLS formation in responsive patients (190).

#### 5.4.5 Chemotherapy and radiotherapy

Chemotherapy induces TME reconstitution, accompanied by B-cell aggregation and TLS formation, thereby enhancing anti-tumor immune efficacy. In esophageal squamous cell carcinoma, studies have shown that the immunosuppressive environment is attenuated during chemotherapy, promoting B-cell maturation and TLS formation (192). In breast cancer, the number of TLSs in tumor cells significantly increased after chemotherapy, with ICOSL+ B-cell subsets interacting with T cells, which significantly improved patient survival rates (80). Similarly, after chemotherapy for hepatoblastoma, large numbers of lymphocytes appeared in tumor cells, inducing immunogenic cell death and initiating an anti-tumor immune response (193).

However, chemotherapy can have side effects that impact TLS maturity, reducing the prognostic value after treatment. For instance, in lung squamous cell carcinoma, it was found that patients who received cortisol treatment to alleviate chemotherapy side effects showed a decreased proportion of TLSs containing germinal centers, indicating weakened TLS maturity and its effect on patient prognosis (14). Thus, the targeted use of chemotherapy-related drugs is necessary to purposefully induce TLS formation and optimize the immunotherapy effect.

Radiotherapy damages tumor cell DNA, leading to cell death; it also modulates the TME, promoting immune cell infiltration (194). For example, in breast cancer samples, radiotherapy was shown to increase TLS apoptosis and induce dynamic changes in immune cell populations (194). However, whether radiotherapy can directly induce TLS formation remains uncertain in current research. Radiotherapy may present a new avenue for inducing TLSs, warranting further investigation.

#### 5.4.6 Other induction methods

Artificial induction of TLS formation in tumor tissue creates a localized immune activation microenvironment. Artificially induced TLSs may enhance treatment effectiveness by increasing immune cell infiltration and activity within tumor tissues. The porous structure of three-dimensional scaffolds can recruit large numbers of immune cells, leading to the formation of artificial TLSs that interact with immune checkpoints to inhibit tumor growth and induce anti-tumor immunity (195). Researchers transplanted collagen sponge scaffolds containing LTα1β2, CCL19, CCL21, CXCL12, CXCL13, and soluble RANK ligands, which over time resulted in the development of immune-competent artificial TLSs with potential applications for cancer treatment (196). Additionally, a study identified a STING activator hydrogel (ZCCG) that effectively enhances immune cell infiltration and the expression of lymphocyte-recruiting chemokines in the TME. This approach promotes TLS formation, improves the therapeutic effect of PD-1 blockade, and inhibits tumor progression, metastasis, and recurrence (197).

## 6 Conclusion and future prospects

With the in-depth study of the immune microenvironment of gastric cancer tumors, the potential role of immunotherapy in treating gastric cancer has become increasingly evident. TLSs have emerged as a promising target for cancer treatment, and their application in clinical immunotherapy for gastric cancer is expanding. B cells located in the germinal centers of TLSs are critical components of the immune system, playing a crucial role in the immune response against gastric cancer. Increasing evidence reveals the key role of TLSs and B cells in tumor development. By assessing the functional status of TLSs and B cells in patients, the immune status of the TME can be modulated to enhance tumor recognition and clearance by the immune system, providing new biomarkers for cancer diagnosis and prognostic assessment.

However, on the path of exploring the application of TLSs-targeted therapies in gastric cancer treatment, there are still many potential challenges and risks. Due to the complexity of the immune system itself and the diversity of TLSs and B cells in the immunoregulatory network, targeted interventions on them may disrupt the original immune balance of the body, leading to adverse consequences such as autoimmune diseases and excessive inflammatory responses. Moreover, the individual differences among patients are extremely large, including the basic immune status and previous disease history. As a result, the same TLSs-targeted treatment regimen may produce completely different effects in different patients, which is undoubtedly a huge challenge for the precise implementation of TLSs-targeted therapies. In addition, there are obvious limitations in the detection and evaluation methods related to TLSs. Although TLSs are known to be of great significance in gastric cancer immunotherapy, there is a lack of specific biomarkers for TLSs and advanced imaging techniques for TLSs detection. The existing detection methods may not be able to accurately distinguish TLSs with different maturities and functional states, resulting in difficulties in effectively stratifying patients and providing personalized treatment. Clinically, it is impossible to accurately determine the quantity, distribution, and internal cell composition of TLSs, making it difficult for doctors to adjust the treatment strategy according to the actual situation of TLSs in cancer patients, thus affecting the precision and effectiveness of TLSs-targeted therapies.

Based on the above challenges and risks, future research directions should focus on several key areas. Firstly, through interdisciplinary research, such as molecular biology and imaging techniques, we can deeply explore biomarkers that can accurately reflect the characteristics of TLSs and develop high-resolution and high-sensitivity imaging technologies to clearly observe the structure, functional status, and dynamic changes of TLSs *in vivo*. Secondly, it is necessary to deeply investigate the underlying causes of immune adverse reactions in different individuals after receiving TLSs and B cell-targeted therapies, and then explore effective preventive and coping strategies. Thirdly, we need to deeply explore the complex interaction mechanisms between TLSs and B cells in the gastric cancer microenvironment, especially their specific manifestations in different gastric cancer subtypes and disease stages, and develop highly efficient and safe targeted therapies accordingly, so as to achieve an effective translation from basic research to clinical applications, truly enabling TLSs and related B cells to realize their potential great value in gastric cancer treatment, and bringing more effective treatment options and better treatment outcomes for gastric cancer patients.
